# PGMF-VINS: Perpendicular-Based 3D Gaussian–Uniform Mixture Filter

**DOI:** 10.3390/s24196482

**Published:** 2024-10-08

**Authors:** Wenqing Deng, Zhe Yan, Bo Hu, Zhiyan Dong, Lihua Zhang

**Affiliations:** 1 Academy for Engineering and Technology, Fudan University, Shanghai 200433, China; 22210860102@m.fudan.edu.cn (W.D.); 22210860063@m.fudan.edu.cn (Z.Y.); 22210860040@m.fudan.edu.cn (B.H.); lihuazhang@fudan.edu.cn (L.Z.); 2 Engineering Research Center of AI and Robotics, Ministry of Education, Shanghai 200433, China

**Keywords:** SLAM, robotic version, location, monocular VIO

## Abstract

Visual–Inertial SLAM (VI-SLAM) has a wide range of applications spanning robotics, autonomous driving, AR, and VR due to its low-cost and high-precision characteristics. VI-SLAM is divided into localization and mapping tasks. However, researchers focus more on the localization task while the robustness of the mapping task is often ignored. To address this, we propose a map-point convergence strategy which explicitly estimates the position, the uncertainty, and the stability of the map point (SoM). As a result, the proposed method can effectively improve the quality of the whole map while ensuring state-of-the-art localization accuracy. The convergence strategy mainly consists of a perpendicular-based triangulation and 3D Gaussian–uniform mixture filter, and we name it PGMF, perpendicular-based 3D Gaussian–uniform mixture filter. The algorithm is extensively tested on open-source datasets, which shows the RVM (Ratio of Valid Map points) of our algorithm exhibits an average increase of 0.1471 compared to VINS-mono, with a variance reduction of 68.8%.

## 1. Introduction

Visual–Inertial SLAM (VI-SLAM) has a wide range of applications spanning robotics, autonomous driving, AR, and VR. Among them, Visual SLAM systems has gained significant interest in recent years compared to Lidar SLAM, owing to their smaller size, lower cost, and convenience. Monocular Visual–Inertial SLAM systems are low-cost, high-precision perception solutions that provide state estimation for navigation tasks in motion. They have great potential due to their advantages of a small size and light weight.

In monocular current VI-SLAM, researchers focus more on the localization task, primarily on how to determine the position and achieve more accurate localization. However, mapping tasks are often overlooked. Researchers often consider mapping completed with a simple triangulation after performing localization tasks, which is insufficient and unreliable. Therefore, we propose a convergence strategy for map points.

Each map point is observed multiple times in a series of camera measurements, so it is crucial to calculate the position in the world coordinate system with multiple observations. Many algorithms directly triangulate multiple observations [1,2]; however, the drawback of this approach is that it does not consider the stability of map points (SoM). In other words, these algorithms triangulate all features observed, even if they are noisy points, mismatched points, or dynamic points. This leads to maps filled with noise and a decrease in pose accuracy.

Therefore, we propose a convergence strategy for map points. It estimates the position and the uncertainty in $R^{3}$ for each map point through a perpendicular-based triangulation. Additionally, we propose a 3D Gaussian–uniform mixture filter based on Bayesian theory, which iteratively fuses multiple observations and explicitly calculates the SoM. These three parameters are updated each time new measurements arrive, and the uncertainty and SoM together determine the convergence of the map points. Specifically, when the uncertainty is sufficiently small and the SoM is sufficiently high, we consider to have obtained an accurate position.

The proposed method is called PGMF-VINS, which has the following contributions:

1. PGMF-VINS includes a convergence strategy for map points, which explicitly estimates the position, the uncertainty, and the stability of map points (SoM). This can effectively improve the quality of the whole map while ensuring state-of-the-art accuracy of pose estimation.
2. The perpendicular-based triangulation and 3D Gaussian–uniform mixture filter are novel theoretical contributions with great potential in SLAM and 3D reconstruction tasks.
3. The algorithm is extensively tested on open-source datasets and shows good performance.

## 2. Related Works

### 2.1. Visual SLAM

The concept of SLAM was first introduced in the mid-1980s, primarily using range-finding sensors like sonar and laser. Visual SLAM emerged later, leveraging advancements in computer vision and camera technology. One of the pioneering works in vSLAM was MonoSLAM [3], which demonstrated real-time 3D mapping and pose estimation using a single camera. This system laid the groundwork for subsequent developments by showcasing the feasibility of using monocular vision for SLAM tasks.

Feature-based methods have been prominent in the development of vSLAM systems. These methods rely on extracting and matching key points (features) from images. Notable algorithms include PTAM [4], which separates tracking and mapping into parallel threads to improve performance. ORB-SLAM2 [5], proposed in 2017, is another significant milestone, utilizing ORB features to achieve real-time performance on monocular, stereo, and RGB-D cameras.

In contrast to feature-based methods, direct methods use the intensity values of pixels directly, avoiding the need for feature extraction and matching. LSD-SLAM [6] is a prominent example, where semi-dense maps are created using direct image alignment techniques. Direct Sparse Odometry (DSO) [7] further advanced this approach by improving accuracy and robustness in challenging environments.

It is important to introduce SVO [8], an algorithm designed for efficient and robust visual odometry. SVO stands out by combining the strengths of both direct and feature-based methods, aiming to achieve high-speed and accurate pose estimation. SVO constructs a sparse map of the environment by selecting key points that are well distributed and have high gradient values through a novel deep filter, and the core idea of this article is inspired by it.

### 2.2. Visual–Inertial SLAM

Vision-only systems have poor performance in extreme situations; Visual–Inertial SLAM (VI-SLAM) is an advanced technique that combines visual and inertial measurements to estimate the pose of a moving device while simultaneously constructing a map of the environment.

In VI-SALM, filter-based approaches play an important role. MSCKF [9] is a pioneering method that shows significant performance gains in robustness and accuracy. It uses an Extended Kalman Filter (EKF) to fuse visual and inertial data. ROVIO [10] incorporates photometric error terms in the EKF framework, improving performance in environments with low-texture or challenging lighting conditions. EKF-based VI-SLAM methods are computationally efficient and suitable for real-time applications. However, they may struggle with long-term accuracy due to linearization errors and limited ability to relocalize.

Optimization-based approaches have become more and more popular in recent years. OKVIS [11] is a tightly coupled method, leveraging keyframe-based nonlinear optimization techniques to refine pose estimates and map features. It performs well in various environments, including those with low texture or dynamic changes. VINS-Mono [1] is a state-of-the-art optimization-based system that has been widely adopted in academia and industry. It features real-time performance on mobile devices and provides robust localization and mapping capabilities in both indoor and outdoor environments. ORB-SLAM3 [2] is an extension of its earlier versions, enhancing their capabilities to provide robust performance in various scenarios. It uses ORB features for reliable key-point detection and matching and employs a keyframe-based strategy that optimizes computational efficiency while maintaining high accuracy in pose estimation and map construction. ORB-SLAM3 has the capability to handle multiple maps simultaneously. SVO2.0 [12] represents a significant advancement in visual odometry, combining the strengths of direct and feature-based methods to achieve robust, real-time performance. Its versatility and efficiency make it a valuable tool for various applications requiring accurate motion estimation and environment mapping. In general, optimization-based methods typically achieve higher accuracy and robustness, particularly in challenging environments with dynamic changes.

The field of VI-SALM has seen significant advancements over the past two decades, driven by innovations in sensor fusion, algorithm design, and computational techniques. The development of robust, real-time systems has paved the way for widespread application in areas such as robotics, augmented reality, and autonomous navigation.

## 3. System Overview

**Figure 1:**
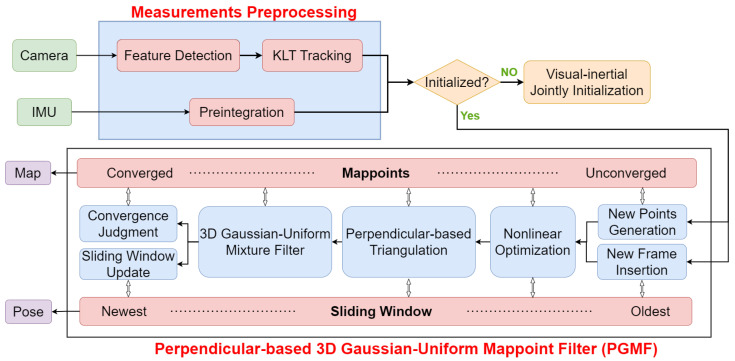
Block diagram illustrating the pipeline of the proposed system. This system consists of three modules: measurements’ preprocessing, visual–inertial joint initialization, and PGMF, with PGMF being the core module.

A block diagram illustrating the pipeline of the proposed system is shown in Figure 1. The system firstly performs measurements’ preprocessing after receiving data from the camera and IMU, in which features are detected and tracked, and IMU measurements of two image frames are preintegrated. Visual–inertial joint initialization is only executed once at the beginning of the entire system to obtain the necessary parameters for subsequent processes. PGMF is the most important and core module. It maintains a sliding window of image frames and the position of map points through three crucial steps: nonlinear optimization, perpendicular-based triangulation, and a 3D Gaussian–uniform mixture filter. Finally, a clean sparse map and accurate up-to-date pose are output in real time.

Compared to state-of-the-art algorithms like VINS [1] and ORB-SLAM [2], which mainly focus on localization tasks and pose accuracy, the proposed algorithm endows the system with the additional capability to obtain stable map points during mapping tasks while maintaining high precision in localization.

It is important to define notations throughout this paper. ${\left(•\right)}^{w}$ means the world coordinates. ${\left(•\right)}^{b}$ is considered as the body coordinates, which are the same as the IMU coordinates. ${\left(•\right)}^{c}$ refers to the camera coordinates. ${\left(•\right)}_{b}^{w}$ represents the transformation from the body coordinates to the world coordinates. Rotation matrices *R* and Hamilton quaternions *q* are equally representing rotation. *P* represents translation. $b_{k}$ and $c_{k}$ represent the body frame and the camera frame while taking the *k*th image, respectively. $g^{w}={\left[0,\;0,\;g\right]}^{T}$ is the gravity in the world coordinates.

### 3.1. Measurements’ Preprocessing

This section preprocesses raw sensor data from the IMU and camera. It is noteworthy that it only tracks data in two consecutive frames for these two types of measurements, aiming for smaller latency and less time consumption.

For visual measurements, when a new image arrives, we first track existing features from the previous frame using the KLT sparse optical flow algorithm [13], then we supplement new features up to a threshold (150–300) by detecting Shi–Tomasi corners [14]. Finally, based on the fundamental matrix, RANSAC is carried out to screen outliers.

For IMU measurements, we use a preintegration algorithm. Preintegration processes the IMU data between two consecutive image frames into a pose increment and a covariance matrix, which does not change with changes in the starting and ending states of the time period. This helps to reduce the computational load. Detailed derivations can be found in [1,15], so we only provide a brief conclusion here:

(1) $$\begin{array}{cc} {\Delta P}_{i,j} & =\sum_{k=i}^{j-1}{\Delta V}_{i,k}\delta t+\frac{1}{2}{\Delta R}_{i,k}\left(a_{k}-{ba}_{k}\right){\delta t}^{2}\\ {\Delta V}_{i,j} & =\sum_{k=i}^{j-1}{\Delta R}_{i,k}\left(a_{k}-{ba}_{k}\right)\delta t\\ {\Delta R}_{i,j} & =\prod_{k=i}^{j-1}Exp\left(\left(\omega_{k}-{bg}_{k}\right)\delta t\right) \end{array}$$

where $\Delta P_{i,j},\Delta V_{i,j},\Delta R_{i,j}$ represent the preintegrations of translation, velocity, and rotation between *i* and *j*, respectively. δt is the time interval between two IMU measurements. $a_{k}$ and $\omega_{k}$ are accelerometer and gyroscope measurements at moment *k*. ${ba}_{k}$, ${bg}_{k}$ are the acceleration bias and gyroscope bias at moment *k*. Exp(•) means the transformation from the axis-angle vector to the rotation matrix. The initial value of the covariance $P_{k+1}$ is set to 0 and is updated according to the following formula: for IMU measurement *k* between *i* and *j*:

(2) $$\begin{array}{cc} P_{k+1} & =\left(I+F_{k}\delta t\;\right)P_{k}{\left(I+F_{k}\delta t\;\right)}^{T}+G_{k}Q{G_{k}}^{T}{\delta t}^{2}\\ F_{k} & =\left[\begin{array}{ccc} \begin{array}{c} 0\\ 0\\ \begin{array}{c} 0\\ 0\\ 0 \end{array} \end{array} & \begin{array}{c} I\\ 0\\ \begin{array}{c} 0\\ 0\\ 0 \end{array} \end{array} & \begin{array}{ccc} \begin{array}{c} 0\\ {-\Delta R}_{ik}{\left[a_{k}-{ba}_{k}\right]}_{\times }\\ \begin{array}{c} -{\left[\omega_{k}-{bg}_{k}\right]}_{\times }\\ 0\\ 0 \end{array} \end{array} & \begin{array}{c} 0\\ -{\Delta R}_{ik}\\ \begin{array}{c} 0\\ 0\\ 0 \end{array} \end{array} & \begin{array}{c} 0\\ 0\\ \begin{array}{c} -I\\ 0\\ 0 \end{array} \end{array} \end{array} \end{array}\right]\\ G_{k} & =\left[\begin{array}{ccc} \begin{array}{c} 0\\ -{\Delta R}_{ik}\\ \begin{array}{c} 0\\ \begin{array}{c} 0\\ 0 \end{array} \end{array} \end{array} & \begin{array}{c} 0\\ 0\\ \begin{array}{c} -I\\ \begin{array}{c} 0\\ 0 \end{array} \end{array} \end{array} & \begin{array}{cc} \begin{array}{c} 0\\ 0\\ \begin{array}{c} 0\\ \begin{array}{c} I\\ 0 \end{array} \end{array} \end{array} & \begin{array}{c} 0\\ 0\\ \begin{array}{c} 0\\ \begin{array}{c} 0\\ I \end{array} \end{array} \end{array} \end{array} \end{array}\right] \end{array}$$

where $\Delta R_{i,k}$ represents the rotation preintegration between *i* and *k*. *I* denotes the 3 × 3 identity matrix. *Q* is a noise covariance matrix $Q=diag(\sigma_{a}^{2},\sigma_{g}^{2},\sigma_{ba}^{2},\sigma_{bg}^{2})$, $\sigma_{a}$ and $\sigma_{g}$ represent the noise of accelerometer and gyroscope measurements, respectively, and $\sigma_{ba}$ and $\sigma_{bg}$ are the random walk noise of the acceleration bias and gyroscope bias. ${\left[•\right]}_{\times }$ means the skew-symmetric matrix of the vector.

### 3.2. Visual–Inertial Joint Initialization

The initialization includes gravity alignment, global scale recovery, and determining the initial pose states and map-point positions. The process is divided into two steps: first, a visual-only SFM is carried out with a set of initial frames, which provides relative poses of these frames in an unknown scale. Then, the first step results are aligned with IMU measurements. This alignment recovers the global scale and solves the gravity vector, ultimately determining the map-point positions. Detailed calculations and derivations can be found in [16].

### 3.3. Sliding Window and Nonlinear Optimization

**Figure 2:**
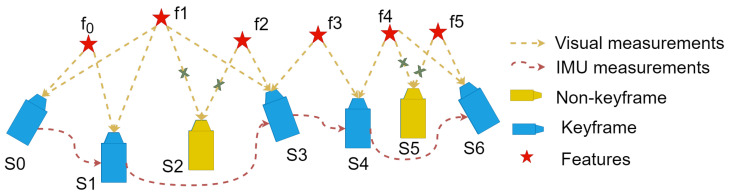
Illustration of the sliding window. The sliding window includes a sequence of frames. A tightly coupled monocular VIO is solved to estimate the state vector of frames. Visual measurements are computed as a reprojection residual, and IMU measurements are computed as a preintegration residual. Non-keyframe are discarded after the optimization.

The illustration of the sliding window is shown in Figure 2. The sliding window includes a sequence of frames. The state vector of these frames is defined as

$$\begin{array}{cc} X & =\left[X_{0},\;X_{1},\;\cdots ,\;X_{n},\;X_{c}^{b},\;P_{0}^{w},\;P_{1}^{w},\;\cdots ,\;P_{m}^{w}\right]\\ X_{k} & =\left[P_{b_{k}}^{w},\;V_{b_{k}}^{w},\;R_{b_{k}}^{w},\;{ba}_{k},\;{bg}_{k}\right],\;\;\;k\;\epsilon \;\left[0,\;n\right]\\ X_{c}^{b} & =\left[P_{c}^{b},\;R_{c}^{b}\right] \end{array}$$

where $X_{k}$ is the IMU state while taking the *k*th image, including position, velocity, orientation, acceleration bias, and gyroscope bias. *n* is total number of frames. $X_{c}^{b}$ is an extrinsic parameter from the camera to the IMU, including translation and rotation. $P_{i}^{w}\left(i\;\epsilon \;\left[0,m\right]\right)$ is the position of features in world coordinates. *m* is total number of map points.

More importantly, the sliding window contains a relationship that maps the visual constraints of the camera from reprojection errors, and the corresponding inertial constraints of the IMU from preintegration errors. The tightly coupled monocular VIO is solved by optimizing these constraints, and the final aim is to minimize the sum of all measurement residuals to obtain a maximum posteriori estimation as

(3) $$\begin{array}{cc} \min_{X}\{\sum_{k\in A}{∥r_{A}\left({\hat{z}}_{b_{k+1}}^{b_{k}},\;X\right)∥}^{2} & +\sum_{i\in B}p\left({∥r_{B}\left({\hat{z}}_{i},\;X\right)∥}^{2}\right)\\ \; & +\sum_{j\in C}p\left({∥r_{C}\left({\hat{z}}_{j},X\right)∥}^{2}\right)\} \end{array}$$

where A is the set of all IMU measurements, and B and C are the set of visual measurements from converged map points and unconverged map points, respectively. For unconverged map points, we construct the reprojection error and simultaneously optimize their positions in world coordinates (a vector of [x, y, z]). However, for converged map points, we do not optimize the positions in world coordinates, as we consider their positions to be reliable and no longer in need of optimization. The advantage of this approach is that it significantly reduces the dimensionality of the state vector during nonlinear optimization, as the number of map points requiring optimization is reduced. Therefore, we separate them into sets B and C to distinguish between them. $r_{A}\left({\hat{z}}_{b_{k+1}}^{b_{k}},\;X\right)$ is the IMU residual. $r_{B}\left({\hat{z}}_{i},\;X\right)$, and $r_{C}\left({\hat{z}}_{j},X\right)$ are visual residuals of their corresponding set. p(•) is the Huber loss function. $∥•∥$ is the Mahalanobis norm.

IMU measurement residuals are computed from preintegration, building upon Equation (1):

(4) $$r_{A}\left({\hat{z}}_{b_{k+1}}^{b_{k}},\;X\right)=\left[\begin{array}{c} \left(P_{j}-P_{i}\right)-{\Delta P}_{i,j}\\ \left(V_{j}-V_{i}\right)-{\Delta V}_{i,j}\\ \begin{array}{c} {R_{i}}^{T}R_{j}{{\Delta R}_{j}^{i}}^{T}\\ {ba}_{j}-{ba}_{i}\\ {bg}_{j}-{bg}_{i} \end{array} \end{array}\right]=\left[\begin{array}{c} {R_{b_{k}}^{w}}^{T}\left(P_{b_{k+1}}^{w}-P_{b_{k}}^{w}-V_{b_{k}}^{w}\Delta t+\frac{1}{2}g^{w}{\Delta t}^{2}\right)-{\Delta P}_{k,k+1}\\ {R_{b_{k}}^{w}}^{T}\left(V_{b_{k+1}}^{w}-V_{b_{k}}^{w}+g^{w}\Delta t\right)-{\Delta V}_{k,k+1}\\ \begin{array}{c} Log\left({R_{b_{k}}^{w}}^{T}R_{b_{k+1}}^{w}{\Delta R}_{k,k+1}^{T}\right)\\ {ba}_{k+1}-{ba}_{k}\\ {bg}_{k+1}-{bg}_{k} \end{array} \end{array}\right]$$

where Δt is the time interval between image moment *k* and k+1. $Log\left(•\right)$ means the transformation from rotation matrix to axis-angle vector.

Visual measurement residuals are divided into two parts based on whether the map points have converged. Their biggest difference lies in the optimization strategy. The position of unconverged map points continue to be estimated in nonlinear optimization, while the converged map points are fixed. However, they compute the residual in the same way, by projecting the map point onto the normalized plane and then subtracting the observation of the *i*th feature $p_{i}$

(5) $$r_{B,\;C}\left({\hat{z}}_{i},X\right)=H\left[{R_{c}^{b}}^{T}\left({R_{b_{k}}^{w}}^{T}\left(P_{i}^{w}-P_{b_{k}}^{w}\right)-P_{c}^{b}\right)\right]-p_{i}$$

where $H\left[•\right]$ means the homogeneous projection. $R_{b_{k}}^{w}$, $P_{b_{k}}^{w}$ is the *k*th pose of the IMU. *k* belongs to the frames that can observe feature $p_{i}$. $P_{i}^{w}$ is the position of feature $p_{i}$ in world coordinates. Using Equation (5), we can derive the Jacobian matrix of the visual residuals with respect to the corresponding variables:

(6) $$\begin{array}{cc} \frac{\partial r_{B,\;C}}{\partial R_{b_{k}}^{w}} & =J_{c}{R_{c}^{b}}^{T}{\left[{R_{b_{k}}^{w}}^{T}\left(P_{i}^{w}-P_{b_{k}}^{w}\right)\right]}_{\times }\\ \frac{\partial r_{B,\;C}}{\partial P_{b_{k}}^{w}} & ={-J}_{c}{R_{c}^{b}}^{T}{R_{b_{k}}^{w}}^{T}\\ \frac{\partial r_{B,\;C}}{\partial R_{c}^{b}} & =J_{c}{\left[{R_{c}^{b}}^{T}\left({R_{b_{k}}^{w}}^{T}\left(P_{i}^{w}-P_{b_{k}}^{w}\right)-P_{c}^{b}\right)\right]}_{x}\\ \frac{\partial r_{B,\;C}}{\partial P_{c}^{b}} & =-J_{c}{R_{c}^{b}}^{T}\\ \frac{\partial r_{B,\;C}}{\partial P_{i}^{w}} & =J_{c}{R_{c}^{b}}^{T}{R_{b_{k}}^{w}}^{T} \end{array}$$

where $J_{c}$ is the Jacobian matrix of $H\left[•\right]$.

The sliding window update handles keyframes and non-keyframes differently. The selection of keyframes follows two criteria: first, the average parallax of tracked features between consecutive frames exceeds a certain threshold, indicating significant movement of the body. Second, to avoid tracking loss in extreme cases, when the number of tracked features falls below a critical value, we treat the new frame as a keyframe. In other situations, the frame is considered as a non-keyframe. Keyframes are retained during updates, while the oldest frame is discarded. For non-keyframes, they are discarded after passing IMU measurements to the next frame.

### 3.4. Convergence Strategy of Map Points

The convergence strategy of map points involves an explicit estimation of the position, the uncertainty, and SoM of each map point in $R^{3}$. These three parameters are updated with each new measurement. The uncertainty and the SoM together determine the convergence of the map point. When the uncertainty is sufficiently small and the SoM is sufficiently high, we consider that an accurate position has been obtained.

The convergence strategy of map points is primarily divided sequentially into a perpendicular-based triangulation and a 3D Gaussian–uniform mixture filter. The perpendicular-based triangulation handles the observation of every feature in the new image. It calculates a position and a variance by combining the latest and the first observations. The specific calculation principles can be found in Section 4. The 3D Gaussian–uniform mixture filter, based on Bayesian theory, stores a state for each map point, including position, variance, and SoM. When receiving the computation result of the previous step, it updates the state of the map point in an iterative manner until the map point converges. The detailed update principles can be found in Section 5.

## 4. Perpendicular-Based Triangulation

Whenever a new image frame arrives, we update the state for each feature in this image. If the ID of the map point is observed for the first time, we create a new map point state. And each time a new image frame arrives, we perform a perpendicular-based triangulation between the current observation and the oldest observation, which ensures that image frames in the middle are not ignored.

Traditional methods have a prerequisite that assumes two observation rays intersect at a definite point in 3D space. However, in reality, we can only obtain an optimal estimate of the camera pose, not the ground truth, which leads to the observation rays intersecting in space in a skewed manner. Our proposed model (perpendicular-based triangulation) addresses this issue and ultimately yields a position and variance in world coordinates. The variance directly represents the error of the current triangulation and on the other hand, implies the uncertainty resulting from an inaccurate pose estimation.

**Figure 3:**
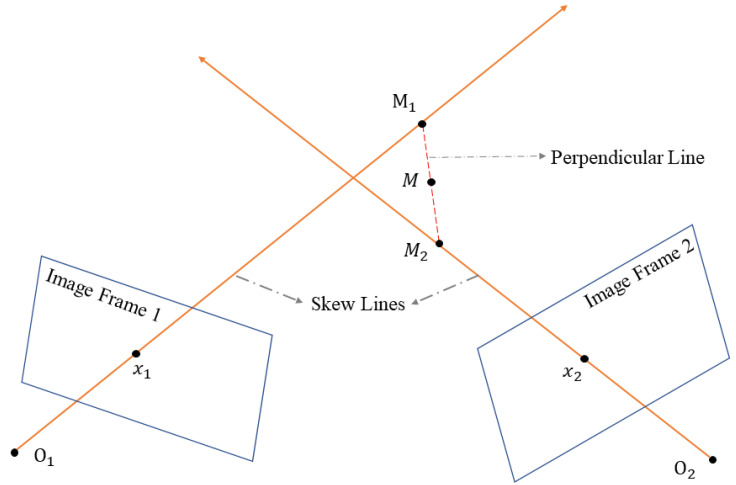
Schematic diagram illustrating the spatial model of perpendicular-based triangulation. $O_{1}$ and $O_{2}$ are the optical centers of image frame 1 and image frame 2, respectively. $x_{1}$ and $x_{2}$ represent the measurements in image coordinates. The observation rays $O_{1}M_{1}$ and $O_{2}M_{2}$ are skew lines and intersect their perpendicular line at $M_{1}$ and $M_{2}$. We consider the real position *M* to be at the midpoint of $M_{1}M_{2}$.

Figure 3 shows the spatial model of the perpendicular-based triangulation. We can observe $x_{1}$ and $x_{2}$ on the image frame. We know the poses of the image frames after nonlinear optimization of the sliding window; let us assume they are $P_{1}^{w}$, $R_{1}^{w}$ and $P_{2}^{w}$, $R_{2}^{w}$. This spatial model follows the following two assumptions:

1. The observation rays $O_{1}M_{1}$ and $O_{2}M_{2}$ are skew lines, due to imprecise poses causing them not to intersect exactly.
2. $M_{1}M_{2}$ is the perpendicular line of the observation rays, and we consider the ground truth of *M* to be at the midpoint of $M_{1}M_{2}$.

Through the spatial model, we know $\vec{O_{1}x_{1}}⊥\vec{M_{1}M_{2}}\to \vec{O_{2}x_{2}}$, that is

(7) $$\begin{array}{cc} \left(R_{1}^{w}\frac{Kx_{1}}{∥Kx_{1}∥}\right)\cdot V_{M_{1}M_{2}} & =\left(R_{2}^{w}\frac{Kx_{2}}{∥Kx_{2}∥}\right)\cdot V_{M_{1}M_{2}}=0\\ x_{1}^{\prime }\cdot V_{M_{1}M_{2}} & =x_{2}^{\prime }\cdot V_{M_{1}M_{2}}=0 \end{array}$$

where *K* represents the camera intrinsic parameters. $V_{M_{1}M_{2}}$ is the unit orientation vector of $M_{1}M_{2}$. $x_{1}^{\prime }$, and $x_{2}^{\prime }$ replace $R_{1}^{w}\frac{Kx_{1}}{∥Kx_{1}∥}$ and $R_{2}^{w}\frac{Kx_{2}}{∥Kx_{2}∥}$. If the depths of $O_{1}M_{1}$ and $O_{2}M_{2}$ are defined as $s_{1}$ and $s_{2}$, we can express $\left(M_{2}^{w}-M_{1}^{w}\right)//M_{1}M_{2}$ as

(8) $$\begin{array}{cc} \left(s_{2}x_{2}^{\prime }+P_{2}^{w}\right)-\left(s_{1}x_{1}^{\prime }+P_{1}^{w}\right) & =cV_{M_{1}M_{2}}\\ s_{2}x_{2}^{\prime }-s_{1}x_{1}^{\prime }+P_{2}^{w}-P_{1}^{w} & =cV_{M_{1}M_{2}} \end{array}$$

where *c* is an arbitrary constant. We multiply both sides of the equation by $x_{1}^{\prime }$ and substitute Equation (7) into Equation (8)

(9) $$s_{2}x_{2}^{\prime }\cdot x_{1}^{\prime }-s_{1}x_{1}^{\prime }\cdot x_{1}^{\prime }+\left(P_{2}^{w}-P_{1}^{w}\right)\cdot x_{1}^{\prime }=cV_{M_{1}M_{2}}\cdot x_{1}^{\prime }=0$$

Similarly, we multiply both sides of the equation by $x_{2}^{\prime }$ and substitute Equation (7) into Equation (8)

(10) $$s_{2}x_{2}^{\prime }\cdot x_{2}^{\prime }-s_{1}x_{1}^{\prime }\cdot x_{2}^{\prime }+\left(P_{2}^{w}-P_{1}^{w}\right)\cdot x_{2}^{\prime }=cV_{M_{1}M_{2}}\cdot x_{2}^{\prime }=0$$

We combine Equations (9) and (10) to form a system of equations:

(11) $$\begin{array}{cc} \; & \left\{\begin{array}{c} \left(x_{1}^{\prime }\cdot x_{1}^{\prime }\right)s_{1}-\left(x_{2}^{\prime }\cdot x_{1}^{\prime }\right)s_{2}=\left(P_{2}^{w}-P_{1}^{w}\right)\cdot x_{1}^{\prime }\\ \left.{\left(x\right.}_{1}^{\prime }\cdot x_{2}^{\prime }\right)s_{1}-\left(x_{2}^{\prime }\cdot 2^{\prime }\right)s_{2}=\left(P_{2}^{w}-P_{1}^{w}\right)\cdot x_{2}^{\prime } \end{array}\right.\\ \Rightarrow & \left\{\begin{array}{c} a_{11}s_{1}-a_{12}s_{2}=b_{1}\\ a_{21}s_{1}-a_{22}s_{2}=b_{2} \end{array}\right.\Rightarrow \left[\begin{array}{cc} a_{11} & -a_{12}\\ a_{21} & -a_{22} \end{array}\right]\left[\begin{array}{c} s_{1}\\ s_{2} \end{array}\right]=\left[\begin{array}{c} b_{1}\\ b_{2} \end{array}\right] \end{array}$$

Equation (11) resembles Ax=b, where the rank of *A* implies different spatial relations. Since the magnitudes of $a_{11}$ and $a_{22}$ are both 1, the rank of *A* cannot be 0. A rank of 1 indicates a special case, where the observation rays $O_{1}M_{1}$ and $O_{2}M_{2}$ are parallel, resulting in infinite solutions for *x*. It is worth noting that this usually implies the body is stationary. A rank of 2 is the most common case, where we can use Cramer’s rule to obtain the unique solution for *x*. Once we get the values of $s_{1}$ and $s_{2}$, we can determine the position of *M*

(12) $$M=\frac{1}{2}\left(M_{1}^{w}+M_{2}^{w}\right)=\frac{1}{2}\left(s_{1}x_{1}^{\prime }+P_{1}^{w}+s_{2}x_{2}^{\prime }+P_{2}^{w}\right)$$

The variance of *M* is divided into $\tau_{a}$ and $\tau_{b}$. $\tau_{a}$ represents the error of the current measurement, which can be obtained by substituting the result of Equation (11) into Equation (8) which is the magnitude of $M_{1}M_{2}$. $\tau_{b}$ represents the components in other directions, for which we take one pixel on the image frame as the error. The specific calculation can be found in [17]. The difference is that the proposed algorithm operates in three dimensions, whereas [17] operates in a two-dimensional plane, so we need to add the $\vec{M_{1}M_{2}}$ vector to calculate. Finally, the total variance is $\tau^{2}=diag\left(\tau_{b}^{2},\tau_{a}^{2},\tau_{b}^{2}\right)$.

## 5. Three-Dimensional Gaussian–Uniform Mixture Filter

Each mappoint is observed multiple times in a series of camera measurements. Therefore, it is crucial to calculate the position of this point in world coordinates with multiple observations. The 3D Gaussian–uniform mixture filter, based on Bayesian theory, continuously integrates new observations in an iterative manner. This method is based on a core assumption: the inliers are considered as camera observation noise, modeled by a three-dimensional Gaussian distribution, while the outliers are represented as white noise, following a uniform distribution, and we represent the joint probability as a Gaussian–uniform mixture distribution, with one key parameter being the SoM. The larger the SoM is, the more likely the point is an inlier; conversely, the smaller the SoM is, the more likely it is an outlier. Ref. [17] discusses the feasibility of this joint probability distribution, and SVO [8] also adopts similar assumptions in its depth filter. We strongly recommend readers to refer to these works for further understanding.

The 3D Gaussian–uniform mixture distribution can be represented as

(13) $$P\left(X_{n}|Z,\pi \right)=\pi N\left(X_{n}|Z,\tau_{n}\right)+\left(1-\pi \right)U\left(X_{n}|S_{b}\right)$$

where *Z* means the ground truth of the position. π represents the probability of being an inlier, while $X_{n}$ and $\tau_{n}$ denote the new measurement values, which are the results of the perpendicular-based triangulation. $N\left(•\right)$ and $U\left(•\right)$ represent Gaussian and uniform distributions in 3D, respectively. $S_{b}$ represents the boundary space of the uniform distribution. We can obtain the recursive form of the probability distribution through Bayesian methods. The specific derivation can be found in [17]. Here, we provide the conclusion:

(14) $$P\left(Z,\pi |a_{n},b_{n},\mu_{n},\Sigma_{n}\right)=P\left(Z,\pi |a_{n-1},b_{n-1},\mu_{n-1},\Sigma_{n-1}\right)\cdot P\left(X_{n}|Z,\pi \right)$$

where $P\left(X_{n}|Z,\pi \right)$ refers to Equation (13). $P\left(Z,\pi |a_{n},b_{n},\mu_{n},\Sigma_{n}\right)=N\left(Z|\mu_{n},\Sigma_{n}\right)\cdot B\left(\pi |a_{n},b_{n}\right)$, which means $P\left(Z,\pi |a_{n},b_{n},\mu_{n},\Sigma_{n}\right)$ is composed of the product of a Gaussian distribution and a Beta distribution. $a_{n}$ and $b_{n}$ in $B(\pi |a_{n},b_{n})$ represent the probabilities for inliers and outliers, respectively, and they jointly determine the calculation of the SoM (π)

(15) $$\pi =\frac{a_{n}}{a_{n}+b_{n}}$$

The issue with Equation (14) is that the product of the two probability distributions on the right-hand side does not strictly equal the probability distribution on the left-hand side. Therefore, we match the first and second moments of the probability distributions on both sides. The moments of the probability distribution on the left-hand side with respect to *Z* and π are

(16) $$\begin{array}{cc} M_{1}\left(Z\right) & =\int_{-\infty }^{\infty }Z\cdot P\left(Z,\pi |a_{n},b_{n},\mu_{n},\Sigma_{n}\right)dZ=\mu_{n}\\ M_{2}\left(Z\right) & =\int_{-\infty }^{\infty }Z^{2}\cdot P\left(Z,\pi |a_{n},b_{n},\mu_{n},\Sigma_{n}\right)dZ=\Sigma_{n}+\mu_{n}{\mu_{n}}^{T}\\ M_{1}\left(\pi \right) & =\int_{-\infty }^{\infty }\pi \cdot P\left(Z,\pi |a_{n},b_{n},\mu_{n},\Sigma_{n}\right)d\pi =\frac{a_{n}}{a_{n}+b_{n}}\\ M_{2}\left(\pi \right) & =\int_{-\infty }^{\infty }\pi^{2}\cdot P\left(Z,\pi |a_{n},b_{n},\mu_{n},\Sigma_{n}\right)d\pi =\frac{a_{n}\left(a_{n}+1\right)}{\left.{\left(a\right.}_{n}+b_{n}\right)\left(a_{n}+b_{n}+1\right)} \end{array}$$

For the right-hand side of Equation (14), we first simplify using Equations (13) and (15)

(17) $$\begin{array}{cc} \; & P\left(Z,\pi |a_{n-1},b_{n-1},\mu_{n-1},\Sigma_{n-1}\right)\cdot P\left(X_{n}|Z,\pi \right)\\ \; & =N\left(Z|\mu_{n-1},\Sigma_{n-1}\right)\cdot B\left(\pi |a_{n-1},b_{n-1}\right)\left\{\pi N\left(X_{n}|Z,\tau_{n}\right)+\left(1-\pi \right)U\left(X_{n}|S_{b}\right)\right\}\\ \; & =\frac{a_{n}}{a_{n}+b_{n}}N\left(Z|X_{n},\tau_{n}\right)N\left(Z|\mu_{n-1},\Sigma_{n-1}\right)B\left(\pi |a_{n-1}+1,b_{n-1}\right)\\ \; & +\frac{b_{n}}{a_{n}+b_{n}}U\left(X_{n}|S_{b}\right)N\left(Z|\mu_{n-1},\Sigma_{n-1}\right)B\left(\pi |a_{n-1},b_{n-1}+1\right) \end{array}$$

We present the product of two Gaussian distributions in three-dimensional space, which serves as a fundamental mathematical result introduced in the derivation process:

(18) $$\begin{array}{cc} N\left(X|\mu_{1},\Sigma_{1}\right)N\left(X|\mu_{2},\Sigma_{2}\right) & =N\left(\mu_{1}|\mu_{2},\Sigma_{1}+\Sigma_{2}\right)N\left(X|\mu^{\prime },\Sigma^{\prime }\right)\\ \Sigma^{\prime }={\left({\Sigma_{1}}^{-1}+{\Sigma_{2}}^{-1}\right)}^{-1} & ,\;\;\;\mu^{\prime }=\Sigma^{\prime }\left({\Sigma_{1}}^{-1}\mu_{1}+{\Sigma_{2}}^{-1}\mu_{2}\right) \end{array}$$

By substituting Equation (18) into Equation (17), we obtain

(19) $$\begin{array}{cc} \; & P\left(Z,\pi |a_{n-1},b_{n-1},\mu_{n-1},\Sigma_{n-1}\right)\cdot P\left(X_{n}|Z,\pi \right)\\ \; & =\frac{a_{n}}{a_{n}+b_{n}}N\left(X_{n}|\mu_{n-1},\tau_{n}+\Sigma_{n-1}\right)N\left(Z|\mu^{\prime },\Sigma^{\prime }\right)B\left(\pi |a_{n-1}+1,b_{n-1}\right)\\ \; & +\frac{b_{n}}{a_{n}+b_{n}}U\left(X_{n}|S_{b}\right)N\left(Z|\mu_{n-1},\Sigma_{n-1}\right)B\left(\pi |a_{n-1},b_{n-1}+1\right)\\ \; & =C_{1}N\left(Z|\mu^{\prime },\Sigma^{\prime }\right)B\left(\pi |a_{n-1}+1,b_{n-1}\right)\\ \; & +C_{2}N\left(Z|\mu_{n-1},\Sigma_{n-1}\right)B\left(\pi |a_{n-1},b_{n-1}+1\right) \end{array}$$

where $\Sigma^{\prime }={\left({\tau_{n}}^{-1}+{\Sigma_{n-1}}^{-1}\right)}^{-1},\;\;\;\;\mu^{\prime }=\Sigma^{\prime }\left({\tau_{n}}^{-1}X_{n}+{\Sigma_{n-1}}^{-1}\mu_{n-1}\right)$. $C_{1}$ is $\frac{a_{n}}{a_{n}+b_{n}}N\left(X_{n}|\mu_{n-1},\tau_{n}+\Sigma_{n-1}\right)$, and $C_{2}$ is $\frac{b_{n}}{a_{n}+b_{n}}U\left(X_{n}|S_{b}\right)$. Since the integral of the probability must be 1, $C_{1}$ and $C_{2}$ need to be normalized, that is, $C_{1}=\frac{C_{1}}{C_{1}+C_{2}}$, $C_{2}=\frac{C_{2}}{C_{1}+C_{2}}$. Then, we compute the moments for *Z* and π in Equation (19)

(20) $$\begin{array}{cc} N_{1}\left(Z\right) & =\int_{-\infty }^{\infty }Z\cdot P\left(Z,\pi |a_{n-1},b_{n-1},\mu_{n-1},\Sigma_{n-1}\right)\cdot P\left(X_{n}|Z,\pi \right)dZ=C_{1}\mu^{\prime }+C_{2}\mu_{n-1}\\ N_{2}\left(Z\right) & =\int_{-\infty }^{\infty }Z^{2}\cdot P\left(Z,\pi |a_{n-1},b_{n-1},\mu_{n-1},\Sigma_{n-1}\right)\cdot P\left(X_{n}|Z,\pi \right)dZ=C_{1}\left(\Sigma^{\prime }+\mu^{\prime }{\mu^{\prime }}^{T}\right)+C_{2}\left(\Sigma_{n-1}+\mu_{n-1}{\mu_{n-1}}^{T}\right)\\ N_{1}\left(\pi \right) & =\int_{-\infty }^{\infty }\pi \cdot P\left(Z,\pi |a_{n-1},b_{n-1},\mu_{n-1},\Sigma_{n-1}\right)\cdot P\left(X_{n}|Z,\pi \right)dZ=C_{1}\frac{a_{n-1}+1}{a_{n-1}+b_{n-1}+1}+C_{2}\frac{a_{n-1}}{a_{n-1}+b_{n-1}+1}\\ N_{2}\left(\pi \right) & =\int_{-\infty }^{\infty }\pi^{2}\cdot P\left(Z,\pi |a_{n-1},b_{n-1},\mu_{n-1},\Sigma_{n-1}\right)\cdot P\left(X_{n}|Z,\pi \right)dZ\\ \; & =C_{1}\frac{\left(a_{n-1}+1\right)\left(a_{n-1}+2\right)}{\left(a_{n-1}+b_{n-1}+1\right)\left(a_{n-1}+b_{n-1}+2\right)}+C_{2}\frac{a_{n-1}\left(a_{n-1}+1\right)}{\left(a_{n-1}+b_{n-1}+1\right)\left(a_{n-1}+b_{n-1}+2\right)} \end{array}$$

By equating the moments of *Z* and π in Equations (16) and (20), we can derive the update formulas for the four parameters $a_{n}$, $b_{n}$, $\mu_{n}$, $\Sigma_{n}$:

(21) $$\begin{array}{cc} a_{n} & =\frac{N_{2}\left(\pi \right)-N_{1}\left(\pi \right)}{N_{1}\left(\pi \right)-N_{2}\left(\pi \right)/N_{1}\left(\pi \right)}\\ b_{n} & =\frac{\left(1-N_{1}\left(\pi \right)\right)\left(N_{2}\left(\pi \right)-N_{1}\left(\pi \right)\right)}{{N_{1}}^{2}\left(\pi \right)-N_{2}\left(\pi \right)}\\ \mu_{n} & =N_{1}\left(Z\right)\\ \Sigma_{n} & =N_{2}\left(Z\right)-\mu_{n}{\mu_{n}}^{T} \end{array}$$

The update formulas for the four parameters in Equation (21) strictly replace the recursive formula based on (14). When the system is running, parameters are updated using explicit analytical expressions, effectively reducing computational load and memory usage, making it a highly efficient method. The convergence judgment is executed after updating the map points, and there are two termination conditions. First, the variance is decomposed using an SVD, and the singular values of the SVD result need to be smaller than a certain threshold (1$\times 10^{-4}$∼1$\times 10^{-2}$). Second, the SoM needs to be greater than a certain threshold (0.40∼0.65). When both conditions are satisfied, we consider this map point as an inlier. It should be noted that if the SoM is less than a certain threshold (0.25∼0.40), we can consider this map point as an outlier and then remove it from the map. This situation may arise due to noise, lighting changes, occlusions, overlaps, and extreme environments.

## 6. Experimental Results

To evaluate the effectiveness and robustness of the VIO system of our proposed method, we conducted a series of experiments. In terms of map quality improvement, we collected data on the recall rate of convergent points and the divergence rate of outliers. Regarding localization accuracy, we tested on the publicly available EUROC MAV dataset [18] and compared the performance with state-of-the-art algorithms. It is worth noting that for the sake of a lightweight system and a clearer evaluation of the proposed method, we did not utilize marginalization, loop detection, or pose relocalization modules.

The experiments were conducted on a desktop PC with an Intel® Core™ i5-12600 CPU @ 3.70 GHz × 8 and 16 GB memory.

### 6.1. Localization Accuracy

**Figure 4:**
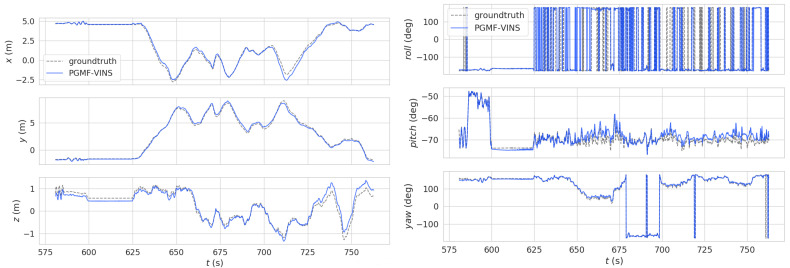
Pose comparison of trajectories. **Left**: position comparison for x, y, z. **Right** orientation comparison for roll, pitch, and yaw.

We used the EVO tool to help process the experimental result data and used images from the left camera. Figure 4 shows the pose comparison of trajectories in the EUROC MH01 sequence. It is evident that PGMF-VINS tracked well in the x, y, and z dimensions. However, in the comparison of orientation data, while the pitch and yaw were still tracked well, the roll direction showed ordinary tracking. The problem was caused by the aggressive movements of the drone in the dataset, which is one of the most difficult challenges; however, our method achieved competitive performance compared to VINS-mono in aggressive movements. Overall, PGMF-VINS performed well in the pose tracking of the localization task.

In Table 1 and Table 2, the RMSEs (Root-Mean-Square Errors) of the RPE (Relative Pose Error) and APE (Absolute Pose Error) for the EUROC dataset are provided. We conducted three tests and took the average of the RPE. Red indicates the best measurement for the sequence, while green indicates a better average value. According to the experimental results, PGMF-VINS achieved the best values for most sequences. In the V1 scene sequences and the difficult sequences of the MH scene, PGMF-VINS performed exceptionally well, indicating its significant potential in more challenging scenarios.

### 6.2. Map Quality Improvement

**Figure 5:**
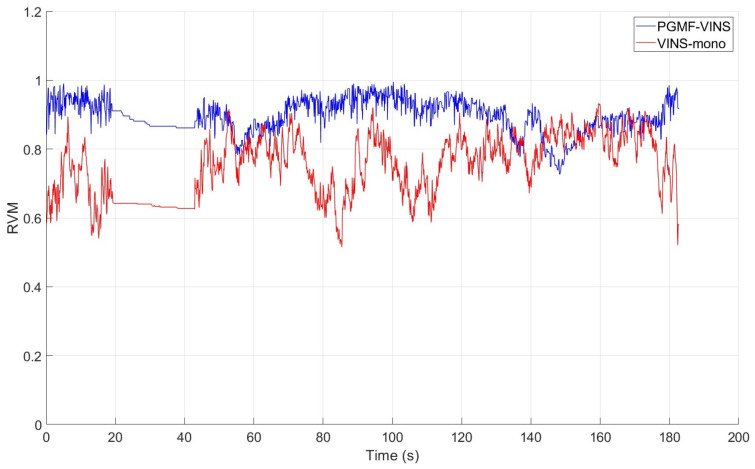
The comparison of the RVM over time.

The core objective of the proposed method was to improve map quality. To evaluate this effect, we collected relevant data on map points and analyzed the recall rate of convergent points and the divergence rate of outliers. We evaluated the proposed algorithm using the Ratio of Valid map points (RVM) metric and compared it with other algorithms to verify the improvement in map quality. The RVM represents the ratio of map points used for pose estimation to the total number of map points. A higher RVM indicates more effective information and better map quality. As shown in Figure 5, the RVM over time clearly demonstrated that the overall performance of the proposed algorithm (PGMF-VINS) surpassed that of VINS-mono. Table 3 presents specific data, showing that the RVM of PGMF-VINS exhibited an average increase of 0.1471 compared to VINS-mono, with a variance reduction of 68.8%.

**Figure 6:**
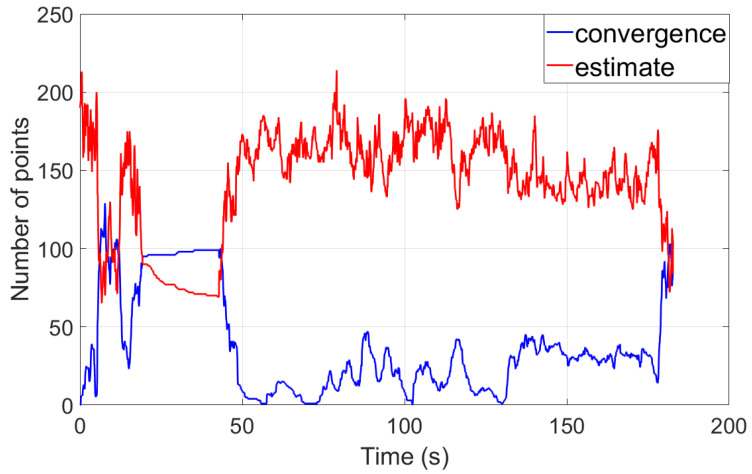
The number of converged and estimated map points within the sliding window.

Figure 6 shows the number of converged and estimated map points within the sliding window. In the first 0∼20 s, the MAV is flying within a small range, so we can see the number of converged map points increasing. From 20∼40 s, the MAV is stationary, so the number of converged map points remains almost unchanged, while the number of estimated map points decreases. From 40∼180 s, the number of estimated map points exceeds the number of converged map points because the MAV is moving forward, meaning that most map points are not sufficiently observed, so the number of converged map points is smaller. During that phase, the number of converged map points fluctuates between 0 and 50.

**Figure 7:**
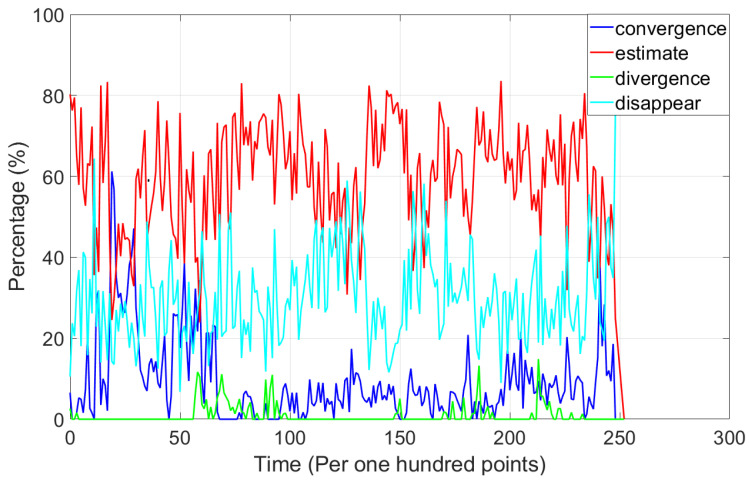
The recall rate per hundred points. The recall rate of convergence, estimation, divergence, and disappearance states for every hundred points.

Figure 7 shows the recall rate per hundred points. We can see the recall rate of convergence, estimation, divergence, and disappearance states for every hundred points over the index of map points. The estimate state was the most common state, indicating that the positions of most points were continuously being estimated. This state fluctuated between 0.4 and 0.8. The percentage of convergence, divergence, and disappearance states validated the effectiveness of the proposed method, as it improved map quality by achieving the following: (1) converging the map points which were observed to be stable; (2) excluding outlier points caused by noise, lighting changes, occlusions, and extreme environments; and (3) filtering out random noise points that appeared occasionally.

**Figure 8:**
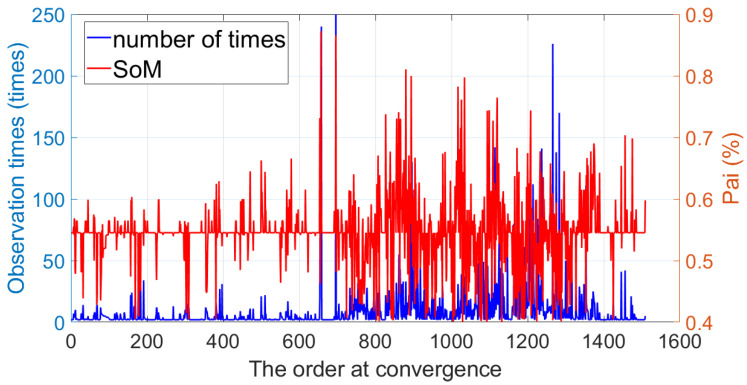
The observation times and SoM at convergence.

Figure 8 illustrates the observation times and SoM at convergence. It can be observed that the majority of points had an SoM value between 0.45 and 0.70 at convergence, indicating they were reliable and stable map points. In our experiments, the SoM of outliers typically fell below 0.4. Additionally, the observation times of convergent map points were mainly distributed in the range [2, 20]. With the image publication frequency manually set to 10 Hz, this implied that the time required for these map points to converge from estimate was controlled within 2 s.

## 7. Conclusions and Future Work

In this paper, we proposed a monocular VI-SLAM method using a novel convergence strategy of map points that could improve the quality of the map. Meanwhile, the proposed method boasted a localization accuracy on par with state-of-the-art algorithms. The convergence strategy was primarily composed of a perpendicular-based triangulation and a 3D Gaussian–uniform mixture filter, and we provided a detailed derivation process for them. Finally, we validated the performance on an open-source dataset by comparing against other state-of-the-art approaches.

We also observe some limitations of the proposed method. In the future, we will differentiate the mapping and localization tasks more clearly and run them in a parallel and tightly coupled manner. Furthermore, the majority of existing SLAM methods assume equal weights for visual measurements during optimization, which is actually unreasonable. Our proposed method provides a way to implement variable weights for visual measurements in the optimization graph, which can be defined from the uncertainty. However, deeper research is still necessary to further improve the accuracy and robustness of the system.

## Figures and Tables

**Table 1:** RMSE of the RPE for the EUROC dataset (full transformation for delta = 1.0 m with SE3 Umeyama alignment).

	PGMF-VINS				VINS-Mono			
	**Times 1**	**Times 2**	**Times 3**	**Average**	**Times 1**	**Times 2**	**Times 3**	**Average**
MH_01_easy	0.119650	0.163103	**0.103939**	0.128897	0.119660	0.119658	0.119670	**0.119663**
MH_02_easy	0.140835	**0.112661**	0.180567	0.144688	0.128412	0.128410	0.128402	**0.128408**
MH_03_medium	0.189257	**0.163397**	0.231937	**0.194864**	0.311137	0.311178	0.311134	0.311150
MH_04_difficult	0.246830	0.207699	**0.197012**	**0.217180**	0.341868	0.360032	0.321771	0.341224
MH_05_difficult	**0.190091**	0.211126	0.241917	**0.214378**	0.295795	0.330839	0.295793	0.307476
V1_01_easy	**0.157847**	0.414040	0.214192	**0.262026**	0.311190	0.311189	0.311203	0.311194
V1_02_medium	**0.194999**	0.262441	/	**0.228720**	0.298319	0.298369	0.298319	0.298336
V1_03_difficult	0.690327	/	0.747513	0.718920	0.623400	/	**0.623400**	**0.623400**
V2_01_easy	1.220313	0.415527	0.745244	0.793695	0.171696	0.171696	**0.171688**	**0.171693**
V2_02_medium	0.704125	0.491096	**0.138500**	0.444574	0.172795	0.192532	0.192529	**0.185952**
V2_03_difficult	/	0.611915	0.868672	0.740294	/	**0.461134**	0.470645	**0.465890**

Note: Red indicates the best, green indicates better average values.

**Table 2:** RMSE of the APE for the EUROC dataset.

	VINS-Mono	MSCKF	SVO	PGMF-VINS (Ours)
MH_01	0.234	0.420	**0.170**	**0.194**
MH_02	0.285	0.450	**0.270**	**0.279**
MH_03	0.867	**0.230**	0.420	**0.371**
MH_04	0.666	**0.370**	1.000	**0.530**
MH_05	0.614	**0.480**	0.600	**0.519**

**Table 3:** Average and standard deviation of the RVM.

Method	Avg.	Std.
VIN-mono	0.7492	0.0077
PGMF-VINS (ours)	0.8963	0.0024

## Data Availability

The original data presented in the study are included in the article.
